# Evaluation of chemosensitivity prediction using quantitative dose–response curve classification for highly advanced/relapsed gastric cancer

**DOI:** 10.1186/1477-7819-11-11

**Published:** 2013-01-22

**Authors:** Teppei Matsuo, Satoshi S Nishizuka, Kazushige Ishida, Fumitaka Endo, Hirokatsu Katagiri, Kohei Kume, Miyuki Ikeda, Keisuke Koeda, Go Wakabayashi

**Affiliations:** 1 Laboratory of Molecular Therapeutics Iwate Medical University School of Medicine, 19-1 Uchimaru, Morioka, Iwate, 020-8505, Japan; 2 Department of Surgery Iwate Medical University School of Medicine, 19-1 Uchimaru, Morioka, Iwate, 020-8505, Japan; 3 MIAST (Medical Innovation by Advanced Science and Technology) Program, Iwate Medical University School of Medicine, 19-1 Uchimaru, Morioka, Iwate, 020-8505, Japan; 4 Institute for Biomedical Sciences, Iwate Medical University, 2-1-1 Nishitokuta, Yahaba, Iwate, 028-3694, Japan

**Keywords:** Gastric cancer, Chemosensitivity, Dose–response curves, Ascites

## Abstract

**Background:**

The use of standard chemotherapy regimens has changed the application of chemosensitivity tests from all chemotherapy-eligible patients to those who have failed standard chemotherapy, which includes patients with highly advanced, relapsed, or chemoresistant tumors.

**Methods:**

We evaluated a total of 43 advanced primary and relapsed gastric cancers for chemosensitivity based on drug dose response curves to improve the objectivity and quality of quantitative measurements. The dose response curves were classified based on seven expected patterns. Instead of a binary chemosensitivity evaluation, we ranked drug sensitivity according to curve shapes and comparison with the peak plasma concentration (ppc) of each drug.

**Results:**

A total of 193 dose response curves were obtained. The overall informative rate was 67.4%, and 85.3% for cases that had a sufficient number of cells. Paclitaxel (PXL)and docetaxel tended to show a higher rank, while cisplatin (CIS) and 5-fluorouracil (5-FU) tended to show resistance, particularly among the 20 cases (46.5%) that had recurrent disease after receiving chemotherapy with CIS and S-1 (5-FU). As such, we speculate that the resistant pattern of the chemosensitivity test suggests that cells with acquired drug resistance were selected by chemotherapy. Indeed, we observed a change in the chemosensitivity pattern of a sample before and after chemotherapy in terms of PXL sensitivity, which was used after primary chemotherapy.

**Conclusions:**

These results suggest that: (i) the dose–response pattern provides objective information for predicting chemosensitivity; and (ii) chemotherapy may select resistant cancer cell populations as a result of the therapy.

## Background

Standard chemotherapy for gastric cancer is considered to have a significant treatment effect as evaluated by disease free survival (DFS) and overall survival (OS) [1-3]. Postoperative chemoradiotherapy for gastric cancer was conducted in the United States with DFS and three-year OS rates of 48% and 50%, respectively [4]. In Europe and the United Kingdom, the result of the MAGIC trial for perioperative chemotherapy was also significant [5]. In Japan, verification of adjuvant chemotherapy with S-1, an oral fluoropyrimidine, found an 80.1% three-year OS and 72.2% DFS, which is now the standard of care for postoperative Stage II/III patients [2]. However, despite the implementation of standard adjuvant chemotherapy, the recurrence rate remains 30% to 40% within five years of therapy [2,5-7]. Hence, highly advanced chemo-naive primary cancers as well as relapsed tumors after adjuvant chemotherapy are major subjects for chemosensitivity tests.

Most previous reports of chemosensitivity assays from the 1990s demonstrated the utility of cell-based assays to aid in the selection of drugs for treating advanced cancers [8-10]. This was useful information for clinicians because there was neither a consensus standard therapy nor large-scale clinical trials established for advanced cancers. With the recent advances in well-controlled large-scale clinical trials, some standard therapies for advanced gastric cancer have been established [1-3]. Accordingly, the indications for chemosensitivity tests have changed in recent times because they are no longer urgently needed for selecting first line therapies. Therefore, the chemosensitivity test has become an approach that can determine appropriate drugs for patients whose treatments fail or who experience recurrence after standard therapies.

Rigid quantitation by conventional chemosensitivity assays has been quite challenging because the number of available tumor cells from solid tumors is extremely limited, which precludes the construction of drug dose–response curves from tumor cells [11,12]. The results from a limited number of data points may not be of sufficient quality for measuring the non-linear associations between drug dose and cell viability. In the present study, chemosensitivity assays for gastric cancers are performed on samples obtained from highly advanced lesions, from which greater numbers of cells can be obtained compared to pre-operative or biopsy specimens, because these larger samples will not affect the definitive pathological diagnosis. We consider the availability of a large number of cells to be a substantial advantage in terms of quantitative measurement of chemosensitivity.

Tumor cells from ascites are generally plentiful and seem to have greater viability in primary culture compared to cells from solid tumors. The availability of large numbers of tumor cells provides the opportunity to quantify tumor cell activity based on dose–response models. Hence, we conducted a chemosensitivity assay that employs tumor classification based on the patterns of dose response curves obtained from multiple drug concentration points derived from a sufficient number of tumor cells. Here we report the results of a chemosensitivity assay with a dose–response curve classification using specimens from highly advanced tumors, with special focus on tumor cells obtained from the ascites of patients with peritonitis carcinomatosa.

## Methods

### Patients

Forty-three gastric cancer patients who had either higher than stage II disease or a recurrence were reviewed (Table 1). Tumor samples were collected from patients after written informed consent was obtained according to Institutional Review Board guidelines (H21-96, Iwate Medical University). The test was approved by the Advanced Medical Care categories of the Japanese Ministry of Labor and Welfare.

**Table 1:** Patient characteristics

**Characteristic**	**Total (number = 43)**		**Ascites (number = 22)**		**Solid tumor (number = 21)**	
	**Number**	**%**	**Number**	**%**	**Number**	**%**
Age, years						
Median	59		53		68	
Range	22 to 85		22 to 78		30 to 85	
Sex						
Male	30	69.8	15	68.2	15	71.4
Female	13	30.2	7	31.8	6	28.6
Stage ^a, b^						
II	2	4.7	0	0	2	9.5
IIIA	1	2.3	0	0	1	4.8
IIIB	5	11.6	1	4.5	4	19.0
IV	35	81.4	21	95.5	14	66.7
Histology^b^						
Intestinal	10	23.3	5	22.7	5	23.8
Diffuse	33	76.7	17	77.3	16	76.2
Stroma^b^						
Medullary type	5	11.6	0	0	5	23.8
Intermediate type	9	20.9	3	13.6	6	28.6
Scirrhous type	12	27.9	6	27.3	6	28.6
Unknown	17	39.5	13	59.1	4	19.0
Pre-therapy						
Performed	30	69.8	16	72.7	14	66.7
Not performed	13	30.2	6	27.3	7	33.3

^a^AJCC (American Joint Commettee on Cancer)/UICC (Union for International Cancer Control) classification.

^b^Stages, Histology, and Stroma for Ascites are of the primary lesions.

### Samples

Specimens from 21 primary solid gastric tumors and 22 ascites samples from peritonitis carcinomatosa patients were obtained. Cells were immediately processed for the chemosensitivity assay or stored at 4°C in serum-free RPMI 1640 medium with heparin for up to six hours.

**Figure 1:**
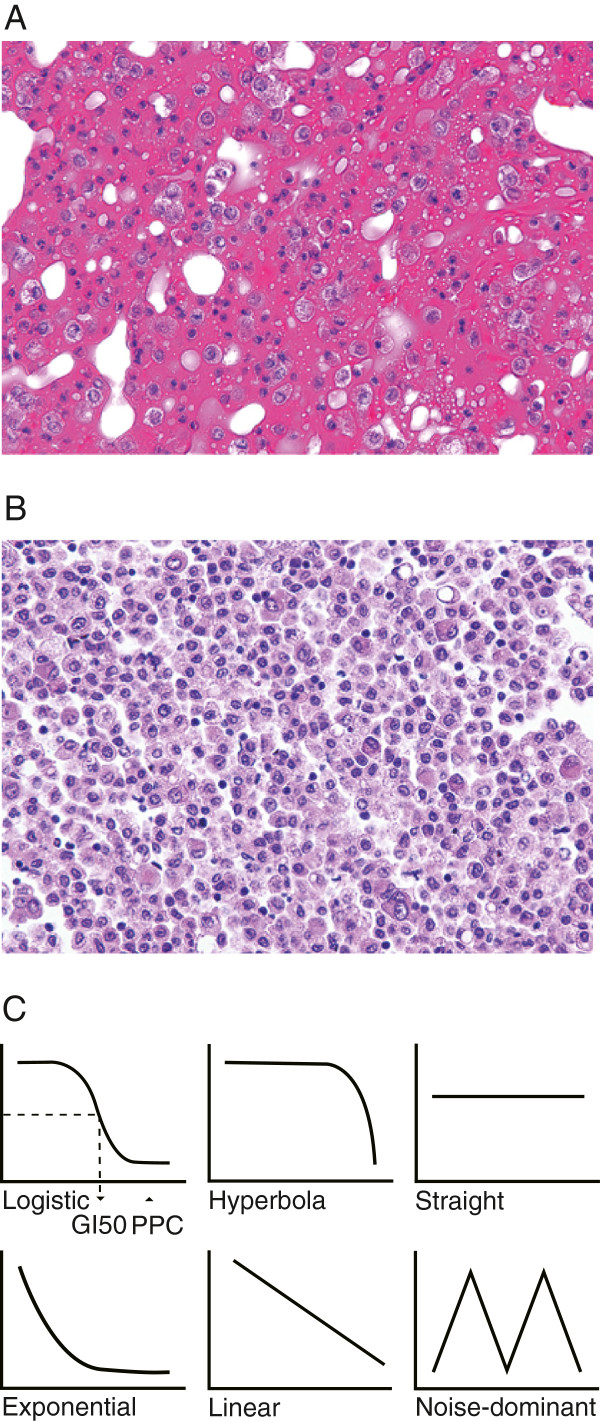
**Epithelial cell enrichment and defined dose–response curve patterns.** Epithelial cell enrichment by density gradient centrifugation. (**A**) Crude cells from ascites taken from a patient with peritonitis carcinomatosa. (**B**) Epithelial cells were enriched by density gradient centrifugation. Six different drug dose–response curve patterns (**C**): The left top panel represents a logistic curve where sample cell growth is dominantly regulated by the drug concentration. The middle top panel represents a hyperbolic curve in which the drug does not suppress cell growth at all practical drug concentrations. Horizontal and vertical axes represent drug concentration and cell viability, respectively.

For primary tumors, specimens were minced and incubated in an enzyme cocktail that included Hank’s balanced salt solution (Sigma-Aldrich Co. LLC, Tokyo, Japan), 7% sodium hydrogen carbonate (Otsuka Pharmaceutical Co. Ltd, Tokyo, Japan), collagenase (Yakult, Tokyo, Japan), deoxyribonuclease I, type IV (Sigma-Aldrich Co.), and protease type XXV (Sigma-Aldrich Co.), with rotation in a 37°C water bath for one hour until the cells were separated [10]. Cells obtained from ascites were centrifuged at 500 x *g* and the resulting cell pellet was then treated with a Ficoll gradient solution to separate the pellet into epithelial and non-epithelial cells (Figure 1). After these steps, both primary and ascites cells were counted and plated into a square flat-bottom 384 microtiter plate at a density of 5,000 to 10,000 cells/well (450 to 900 cells/mm^2^) in serum-free RPMI 1640 in a total volume of 70 μl. Cells were incubated for at least 24 hours before drug administration.

### Anticancer agents

Fourteen anticancer agents were used in the assay (Table 2). The starting concentration of all drugs was 1% of the original vial (actual molar concentrations were different). Each drug was added in a 10-fold dilution series to confirm cell viability as a function of drug dose. The peak plasma concentration (ppc) was used as a reference in comparison with the 50% growth inhibitory concentration (GI_50_: concentration of a drug for which growth is reduced by 50% compared to the untreated control) value, defined as the absorbance of water-soluble tetrazolium (WST) salt, (Dojindo, Mashiki, Japan) at which the growth was reduced by 50% compared to the untreated control [13-15]. Each drug has a unique ppc value, which is the indicator for evaluating whether the effective drug concentration is achievable in the human body. If the ppc is greater than the GI_50_, then the drug is likely to affect cancer cell growth *in vivo*.

**Table 2:** Drugs used in chemosensitivity tests

**Abbreviation**	**Drug**	**Mechanism of action**	**Starting concentration (μM)**	**ppc (μM)**
CIS	cisplatin	DNA synthesis inhibition	1.67 × 10	8.33
5FU	5-fluorouracil	Antimetabolite	3.84 × 10^3^	1.17 × 10^2^
CPT	irinotecan	Topoisomerase I inhibition	2.95 × 10^2^	N.D.
SN38	SN-38	Topoisomerase I inhibition	1.02 × 10^2^	5.10 × 10^-2^
DTX	docetaxel	Microtubule inhibition	1.16 × 10^2^	2.32
PXL	paclitaxel	Microtubule inhibition	7.03 × 10	5.86
MTX	methotrexate	Antimetabolite	5.50 × 10	3.30 × 10
DXR	doxorubicin	DNA synthesis inhibition	1.72 × 10^2^	0.62
EPI	epirubicin	DNA synthesis inhibition	1.72 × 10^2^	1.72 × 10
EPS	etoposide	Topoisomerase II inhibition	3.40 × 10^2^	1.69 × 10
GEM	gemcitabine	Antimetabolite	3.34 × 10^3^	7.34 × 10
OXP	oxaliplatin	DNA synthesis inhibition	1.26 × 10^2^	2.27
VIN	vinorelbin	Microtubule inhibition	9.3 × 10	0.93
LEU	leucovorin	Anti metabolite	5.9 × 10	6.84

N.D., not determined; ppc, peak plasma concentration.

### Growth suppression assay

After 24 hours of drug exposure, 10% medium volume (7 μl) of WST was added to each well of the primary culture and incubated for 3 to 6 hours at 37°C supplemented with 5% CO_2_. Absorbance was measured at 450 nm using a microplate reader (Tristar LB941, Berthold Technologies GmbH & Co. KG, Bad Wildbad, Germany).

### Interpretation of dose–response curves

**Figure 2:**
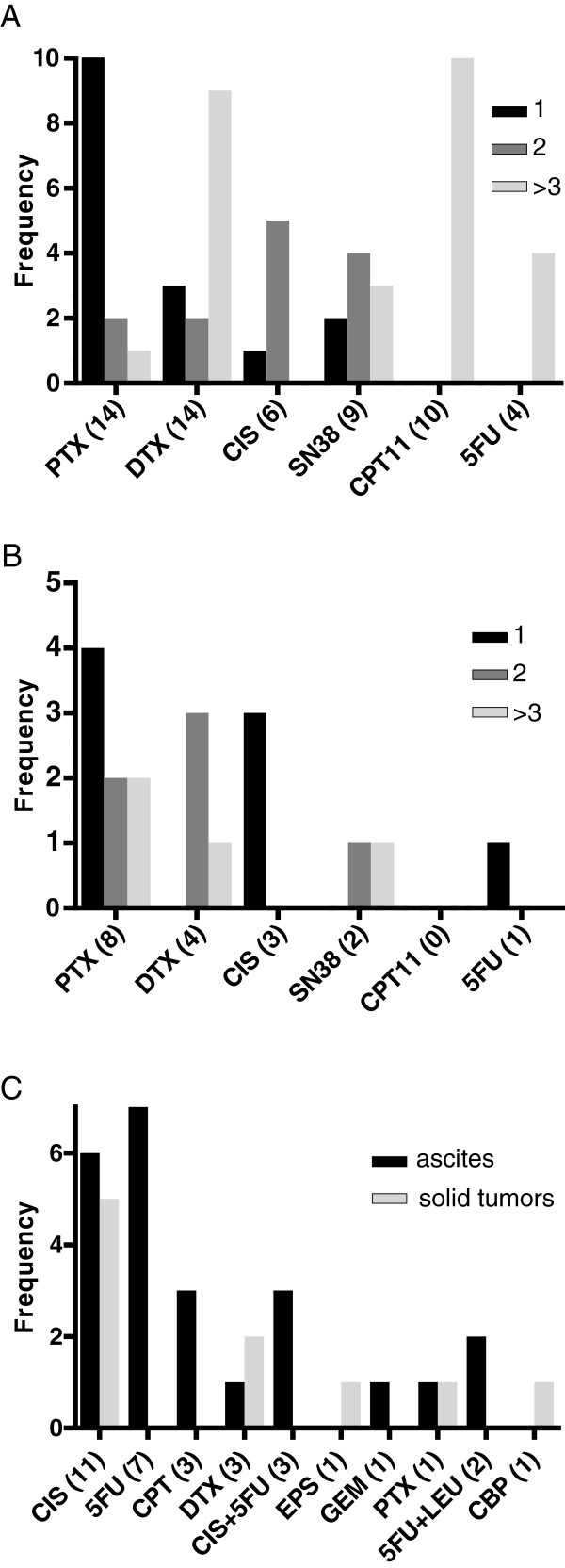
**Chemosensitivity rank. Frequency of drug expectation rank based on GI**_**50**_**.** (**A**) Ascites and (**B**) Solid tumors. (**C**) Frequency of drug resistance defined by a hyperbolic pattern of dose–response curves. GI_50,_ concentration of a drug for which growth is reduced by 50% compared to the untreated control.

To facilitate the objective interpretation of dose–response curves, we defined seven patterns that can be considered to reflect the respective biological properties of primary cultured cancer cells (Figure 2). When the curve is drug concentration-dependent in a logistic fashion, the GI_50_ can be readily calculated as a logistic pattern. When cell viability does not change across all concentrations except for a steep drop at the highest dose, it is considered ‘hyperbolic’, which is a good indicator of drug resistance. Other dose–response curves were eliminated from the chemosensitivity evaluation, but categorization facilitates diagnoses based on the curve-fitting concept. For logistic curves, the GI_50_ can be compared with the ppc of each drug to determine the chemosensitivity rank. The rank is a relative ordering of the assay results in a given sample and provides an idea of which drug is likely to have efficacy towards the tumor. The curve fitting was performed using a non-linear multiple regression analysis with Prism software (Version 5.01, GraphPad Software, Inc., La Jolla, CA, USA).

### Evaluation of chemotherapeutic effect

For those samples where related information was available, the chemotherapeutic effect on tumors in ascites was evaluated by tumor markers, ascites volume, and dietary intake. Although these evaluations are semi-quantitative and often subjective, they may still be informative in terms of the chemotherapeutic effect. The qualitative evaluation criteria for pre- and post-treatment tumor markers are as follows: Up, the marker value is beyond the upper limit of the normal range from within or below the normal range; Stable, the tumor marker value remains below the upper limit of the normal range; and Down, the marker value goes below the upper limit of the normal range from above the normal range. Ascites volume was classified into the following three groups: Up, Stable, and Down, which were evaluated based on comprehensive clinical findings including physical examination, ultrasonography, and computed tomography. Dietary intake was classified into Up, Stable, and Down categories by medical interview, impression of health professionals, and chart review.

## Results

### Chemosensitivity test

A total of 43 gastric cancer specimens, including 22 from ascites and 21 from solid tumors, were examined. A sufficient number of cells for the WST assay was obtained in 34/43 (79%) cases, of which 16/22 (72%) cases were from solid tumors and 18/21 (86%) were from ascites. Only one out of 16 (6%) ascites cases was not analyzed due to low viability, while four out of 18 (22%) solid tumors were not analyzed due to fungal contamination (two cases), bacterial contamination (one case), and low viability (one case). Cells from each tumor sample were plated into wells of a 384-well microtiter plate and subsequently assayed with 3 to 16 drugs individually, each of which provided a dose–response curve. Overall, 29 samples produced 193 dose–response curves with one curve per drug per specimen (Table 3). In ascites cases, we obtained 110 curves from 15 cases (7.3 drugs per specimen) and for solid tumor cases, 83 curves from 14 cases (5.9 drugs per specimen).

**Table 3:** Frequency of curve types

**Curve Types**^**a**^						
**Drug**	**Logistic (%)**	**Hyperbolic (%)**	**Straight (%)**	**Exponential (%)**	**Linear (%)**	**Noise-dominant (%)**
CIS	9(9.7)	11(33.3)	0	1(50.0)	3(23.1)	5(11.6)
5FU	5(5.4)	7(21.2)	3(33.3)	0	3(23.1)	11(25.6)
CPT	10(10.8)	3(9.1)	1(11.1)	0	1(7.7)	5(11.6)
SN38	11(11.8)	0(0.0)	0	0	2(15.4)	8(18.6)
DTX	18(19.4)	3(9.1)	1(11.1)	1(50.0)	0	5(11.6)
PXL	22(23.7)	1(3.0)	1(11.1)	0	1(7.7)	4(9.3)
OXP	3(3.2)	0(0.0)	0	0	0	0
EPS	3(3.2)	1(3.0)	0	0	0	0
GEM	1(1.1)	1(3.0)	0	0	1(7.7)	0
5FU + LEU	0(0)	2(6.1)	0	0	1(7.7)	0
CIS + 5FU	4(4.3)	3(9.1)	1(11.1)	0	0	1(2.3)
MTX	3(3.2)	0	0	0	1(7.7)	3(7.0)
5FU + MTX	1(1.1)	0	1(11.1)	0	0	0
EPI	0	0	1(11.1)	0	0	0
CBP	0	1(3.0)	0	0	0	0
5FU + DTX	1(1.1)	0	0	0	0	0
5FU + SN38	1(1.1)	0	0	0	0	0
DXR	1(1.1)	0	0	0	0	0
VIN	0	0	0	0	0	1(2.3)
Curve Fraction^b^	93(48.2)	33(17.1)	9(4.7)	2(1.0)	13(6.7)	43(22.2)

^a^% indicates fraction per curve type; ^b^% indicates fraction of all 193 curve types.

### Interpretation of dose–response curve data

Each dose–response curve was categorized into one of the six previously determined patterns. Two major patterns (logistic and hyperbolic) represented 65% (126/193) of all patterns. Logistic and hyperbolic patterns are considered to be indicative of ‘drug-dependent cellular viability’ and ‘drug resistance’, respectively. In fact, 24.2% of hyperbolic curves were from tumors that had been previously treated with the corresponding drug. While we did not use curves from other categories (67/193, 35%) for further analysis, they were useful for interpreting curves and in particular helped minimize variations in interpretations by the examiner.

The curves were further evaluated to rank the drugs based on GI_50_ and ppc. From logistic curves, we calculated GI_50_ values to represent how effective the drug was on the cells. GI_50_ values from 93 logistic curves were compared with the corresponding drug’s ppc. Eighty-one out of 93 (87%) curves showed that the ppc was greater than the GI_50_. Based on the fact that the greater the difference between the ppc and GI_50_, the better the chance that the drug is effective, we ranked the set of drugs used for each assay.

**Figure 3:**
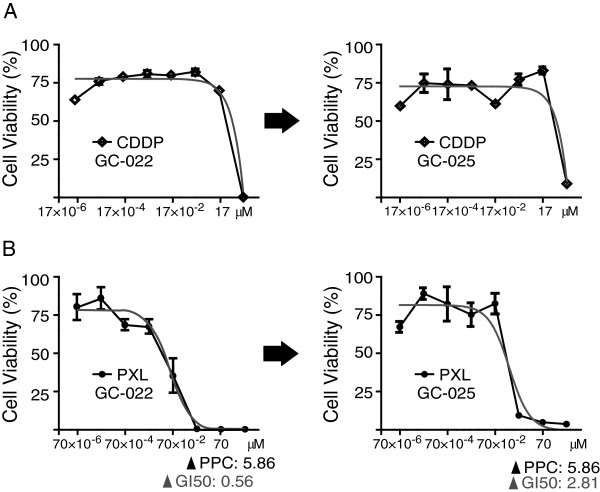
**Replacement of cellular population by chemotherapy.** Drug dose–response curves from a pair of samples from an individual patient who received two chemosensitivity tests, pre- and post-chemotherapy for CIS (**A**) and PXL (**B**).

Although the number of drugs tested for one sample was inconsistent because the number of available cells varied, paclitaxel (PXL) seemed to be most effective (rank, 1), followed by docetaxel (DTX) (rank, 2) and cisplatin (CIS) (rank, 3; Figure 3A and B). PXL showed the highest average rank (rank, 1.29) for ascites (Figure 3A) while CIS (rank, 1; Figure 3B) was the highest for solid tumors.

### Patient outcomes and chemosensitivity test

The number of evaluable cases was 15 for ascites. Two out of the 15 cases were eliminated because of a marked decline in general condition. The remaining 13 cases proceeded to systemic chemotherapy based on the results of the chemosensitivity test. Thus, all chemosensitivity evaluations for 13 cases were based on drugs with a rank of 1 or 2 (Table 4). Tumor markers were decreased or stable in 7/13 (54%) and 11/13 (86%) patients for carcinoembryonic antigen (CEA) and cancer antigen 19–9 (CA19-9), respectively. Ascites volume showed a substantial decrease or remained stable in 11/13 (85%) cases, indicating that ascites volume may be one of the most immediately responsive markers in highly advanced cases. Although dietary intake is less objective than other parameters, increased intake for 8/13 (62%) cases suggests a direct association with the reduction of gastrointestinal burden and minimum adverse effects by the selected chemotherapy.

**Table 4:** Evaluation of chemotherapy by tumor marker, ascites volume, diet

**Tumor Markers**	**Cases**	**%**	**Responded**
CEA			
Up	6	(46%)	
Stable	3	(23%)	54%
Down	4	(31%)	
CA19-9			
Up	2	(15%)	
Stable	7	(54%)	85%
Down	4	(31%)	
Ascites volume			
Up	2	(15%)	
Stable	3	(23%)	85%
Down	8	(62%)	
Diet			
Up	8	(62%)	62%
Stable	0	(0%)	
Down	5	(38%)	

### Replacement of cellular population after chemotherapy

Although the number is limited, we had a pair of samples for which the patient received two chemosensitivity tests, pre- and post-chemotherapy. Case GC-022 showed CIS resistance (hyperbolic), while PXL ranked first in the first chemosensitivity assay at pre-chemotherapy. Ascites volume was increased nine weeks after completion of six cycles of 80 mg/m^2^ PXL weekly. A subsequent chemosensitivity assay (GC-025) on ascites demonstrated a more than five-fold increase in GI_50_ PXL, but they remained resistant to CIS (Figure 3).

## Discussion

The utility of cell-based assays for evaluating chemosensitivity depends on the quality of the quantitative processes, because the number of cells, replicates, and dilution series are critical for obtaining good measurement quality [8-10]. The 384-well microtiter plate used in the present study requires 5,000 to 10,000 cells per well, which allowed us to assess the differences and similarities in drug responses using multiple drug dose points. The drug concentration-cell growth (that is, dose–response) curve is generally not linear, and ideally should be logistic. To evaluate drug sensitivity, the curve must show a logistic curve that is considered to be a pharmacological drug response [12,15]. We confirmed that hyperbolic dose–response curves are a sufficient indicator of drug resistance. Other curve types do not directly indicate any clinical applications, but did help with assay evaluation by multiple examiners in daily practice.

We determined the chemosensitivity rank based on response expectancy instead of binary nominal sensitivity (that is, sensitive or non-sensitive) [8-10]. Although the rank depends on how many drugs are tested in a given assay, PXL seems to be the most frequently highly-ranked drug. CIS and 5-FU (that is, S-1, an oral fluoropyrimidine), which had been the first choice for highly advanced gastric cancer chemotherapy, seemed to have lower ranks in post-treatment tumors, implying that the tumors may have acquired drug resistance during primary chemotherapy. In primary chemo-naive tumors, however, CIS showed good sensitivity, suggesting that CIS is a reasonable choice as a first line drug for primary tumors, as most regimens include this drug.

Although the number of cases is still limited, one of the major benefits of the present approach is the ability to see population changes in cancer cells at different time points for a given patient. For instance, GC-022 and GC-025 are samples from a patient who was treated with six cycles of 80 mg/m^2^ PXL weekly and showed a five-fold increase in GI_50_ values for PXL, while remaining resistant to CIS. It is reasonable to speculate that the acquisition of drug resistance would be due to changes in the tumor cell population [14]. Recent reports demonstrated that genome-wide genetic and epigenetic events accumulate during cancer progression [16]. Therefore, the phenotypic change we observed in the present case suggests that drug-induced phenotypic or genetic/epigenetic changes occurred during our routine cancer therapies [14].

The response rate of standard cancer therapies is still generally around 30% to 40% [1-3]. To determine standard therapies based on tumors of origin through epigenetic studies, large-scale clinical trials are important. However, continued efforts are necessary to minimize the fraction of patients who would not benefit from the standard therapy as well as to provide justification for each type of therapy. The biological properties of cancer cells are often drastically different depending on each patient, so treatments should be flexible based on tumor characteristics. Patients with ascites have very poor life expectancy from the date of diagnosis and many fail either standard or other subsequent therapies [17]. Our chemotherapeutic response expectation rankings suggest that it would be possible to make better drug choices that may prolong survival and significantly decrease the cancer burden for these patients [11].

## Conclusions

Our chemosensitivity assay is still a conventional cell-based technique. However, the assay can more accurately determine appropriate drug regimens based on the tumor response to drugs using a currently available and reliable technique with a sufficient number of tumor cells. The chemosensitivity assay is more applicable to many types of chemotherapeutic drugs. Our present data provide clues for profiling individual tumors based on functional drug response information. As such, data accumulation and acquisition of supporting molecular evidence is warranted.

## Abbreviations

CA19-9: cancer antigen 19–9; CEA: carcinoembryonic antigen; CIS: cisplatin; CPT: irinotecan; DFS: disease free survival; DXR: doxorubicin; DTX: docetaxel; EPI: epirubicin; EPS: etoposide; GEM: gemcitabine; GI_50_: 50% growth inhibitory concentration; LEU: leucovorin; MTX: methotrexate; N.D.: not determined; OS: overall survival; OXP: oxaliplatin; ppc: peak plasma concentration; PXL: paclitaxel; SN38: SN-38; VIN: vinorelbin; WST: water-soluble tetrazolium; 5FU: 5-fluorouracil.

## Competing interests

The authors declare that they have no competing interests.

## Authors’ contributions

TM carried out the chemosensitivity assay and drafted the manuscript; SSN carried out the experimental design and drafted the manuscript; KI carried out the chemosensitivity assay and experimental design; FE carried out the chemosensitivity assay; HK carried out the chemosensitivity assay; KKu carried out the chemosensitivity assay; MI carried out the chemosensitivity assay and experimental design; KKo carried out the experimental design; GW carried out the experimental design. All authors read and approved the final manuscript.
